# Utilization of chitosan/Ag bionanocomposites as eco-friendly photocatalytic reactor for Bactericidal effect and heavy metals removal

**DOI:** 10.1016/j.heliyon.2019.e01980

**Published:** 2019-06-25

**Authors:** Al-Sayed A. Al-Sherbini, Hala E.A. Ghannam, Gamal M.A. El-Ghanam, Amr. A. El-Ella, Ahmed M. Youssef

**Affiliations:** a Department of Measurements, Photochemistry and Agriculture Applications, National Institute of Laser Enhanced Science (NILES), Cairo University, Giza, Egypt; b Pollution Laboratory, National Institute of Oceanography and Fisheries, Alexandria, Egypt; c Packaging Materials Department, National Research Centre, 33 El Bohouth St. (former El Tahrir st.), Dokki, Giza, P.O. 12622, Egypt

**Keywords:** Materials science, Chemistry, Environmental science, Antibacterial activity, Heavy metal removal, Chitosan, Photo-oxidation, Silver nanoparticles

## Abstract

Chitosan is a nontoxic, eco-friendly, and biocompatible natural polymer which could be used in an extensive range of applications for example in the areas of membranes, biomedicine, hydrogels, wastewater treatment, food packaging. Moreover, chitosan based nanomaterials had high sorption capacities, chelating activities, stability, and versatility, that would be potentially applied as green reactants in various scientific and engineering applications. The current study involved the preparation of silver nanoparticles incorporated into chitosan thin films and used for various purposes including photo-oxidation of organic pollutants, heavy metal removal (Cd, Pb, Cr, and Fe) and antibacterial activity. The fabricated chitosan/silver (CS/Ag) bionanocomposites thin films were characterized by the ultraviolet-visible (UV-Vis) spectroscopy, transmission electron microscopy (TEM), and Fourier transforms infrared (FT-IR) spectroscopy. Furthermore, the prepared CS/Ag bionanocomposites had revealed good photodegradation rate, heavy metals removal and antimicrobial activity against gram-negative bacteria like *E. coli*, and gram-positive bacteria like *G. bacillus,* with increasing the loading of different concentrations of chitosan and silver nanoparticles incorporated into the prepared bionanocomposite thin films. Consequently, the prepared CS/Ag bionanocomposites are considered good candidates for wastewater treatment through photo-oxidation of organic pollutants, heavy metal removal as well as respectable antibacterial materials.

## 1 Introduction

The term pollution is defined as the contamination of air, water or soil with one or more material that detracts from its ability to support the original ecosystem or to provide some human needs otherwise used (e.g., Arabian and TiO_2_). In this context, it is well known that water pollutions is one of the most common problems facing our societies due to the charging of the environment with multitude of potentially toxic agents (Ali et al., 2018a,b; Nauman et al., 2018; Youssef and Malhat, 2014; Youssef et al., 2016a,b). Therefore, studying water pollution sources and how to prevent or reduce these sources had the attention of the researchers all over the world. Applying the traditional water treatment processes including phase transfer, biological treatment, thermal, and catalytically oxidation had been also tested. Also, chemical treatment using chlorine, potassium permanganate, ozone, hydrogen peroxide and high energy ultraviolet light was one of the most attractive points for research (Mukherjee and Ray, 1999; El-Nahrawy et al., 2016; Ali et al., 2017; Ali et al., 2018a,b; El-Maghrabi et al., 2018; Youssef et al., 2019a).

In many previous studies a practical limitation in case of these approaches used specifically when the separation of the suspended catalyst from the process is carried out rather than the using of the catalyst as integrated techniques to remove the heavy metal or disinfections. To decrease the cost of the method, the nanocomposites should be supported with a suitable substrate and many trails carried out to use polymers as substrates to improve the treatment processes. So, chitosan as a natural polysaccharide-based biopolymer has become a cornerstone in various scientific and engineering applications (Kumar, 2000; Youssef et al., 2014; Youssef et al., 2015; Youssef et al., 2016a, b; Youssef et al., 2018).

The presence of readily functionalizable hydroxyl and amino groups on the chitosan, and its insolubility in organic solvents make it very attractive in the applications (Chtchigrovsky et al., 2009; Kumar et al., 2015; Haider et al., 2016; Youssef et al., 2019b). The unique properties of chitosan also enhanced its applicability for further functionalization and immobilization of metal complexes through covalent attachment (Kumar et al., 2015; Kamal et al., 2016) and adsorption (Sun et al., 2011; Abd El-Aziz et al., 2019). In addition, chitosan was employed to immobilize photocatalysts for easy recovery as well as enhancement of photodegradation (Mansur et al., 2014; Khoza and Nyokong, 2015). Correspondingly, Chitosan can also be used as a supportive material for metal nanoparticles and it has antimicrobial activities (Mi et al., 2002; Hardy et al., 2004; Youssef et al., 2018). In recent years, much attention has been paid to metallic nanoparticles which exhibit novel optical, electronic, magnetic, and chemical properties owing to their extremely small dimensions and their high surface-to-volume ratio (Wang et al., 2009; Youssef and El-Sayed, 2018). Metal can be extremely toxic to most bacteria and yeast at exceptionally low concentrations. Because of this biocide activity, some particular metals have been used as antimicrobial agents since ancient times. For instance, vessels made of Cu and Ag have been used for water disinfection and food preservation since the time of the Persian kings (Lemire et al., 2013; El-Shibiny et al., 2018).

The aim of the present study is to design of CS/Ag bionanocomposites thin films and be used as integrated technique for photo-oxidation, antimicrobial and heavy metals removal from waste water.

In many previous studies, a practical limitation in the case of these approaches used specifically when the separation of the suspended catalyst from the process is carried out rather than the using of the catalyst as integrated techniques to remove the heavy metal or disinfection. To decrease the cost of the method, the nanocomposites should be supported with a suitable substrate and many trials carried out to use polymers as substrates to improve the treatment processes. So, chitosan as a natural polysaccharide-based biopolymer has become a cornerstone in various scientific and engineering applications (Kumar, 2000; Youssef et al., 2014; Youssef et al., 2015; Youssef et al., 2016a,b; Youssef et al., 2018). The presence of readily functionalizable hydroxyl and amino groups on the chitosan, and its insolubility in organic solvents make it very attractive in the applications (Chtchigrovsky et al., 2009; Kumar et al., 2015; Haider et al., 2016; Youssef et al., 2019b). The unique properties of chitosan also enhanced its applicability for further functionalization and immobilization of metal complexes through covalent attachment (Kumar et al., 2015; Kamal et al., 2016) and adsorption (Sun et al., 2011; Abd El-Aziz et al., 2019). Besides, chitosan was employed to immobilize photocatalysts for easy recovery as well as enhancement of photodegradation (Mansur et al., 2014; Khoza and Nyokong, 2015). Correspondingly, Chitosan can also be used as supportive material for metal nanoparticles and it has antimicrobial activities (Mi et al., 2002; Hardy et al., 2004; Youssef et al., 2018).

In recent years, much attention has been paid to metallic nanoparticles which exhibit novel optical, electronic, magnetic, and chemical properties owing to their extremely small dimensions and their high surface-to-volume ratio (Wang et al., 2009). Metal can be extremely toxic to most bacteria and yeast at exceptionally low concentrations. Because of this biocide activity, some particular metals have been used as antimicrobial agents since ancient times. For instance, vessels made of Cu and Ag have been used for water disinfection and food preservation since the time of the Persian kings (Lemire et al., 2013; El-Shibiny et al., 2018).

The present study aims to design of CS/Ag bionanocomposites thin films and be used as an integrated technique for photo-oxidation, antimicrobial and heavy metals removal from wastewater.

## 2 Materials and methods

### 2.1 Materials

Hydrochloric Acid (HCl), Aldrich, (Puriss), sodium hydroxid (NaOH),Aldrich, (Puriss), trisodium citrate, (C_6_H_5_O_7_Na_3_), Aldrich, (Puriss), silver nitrate (AgNO_3_), Aldrich, (Puriss), polyvinylidene chloride (PVDC) is a homo-polymer of vinylidene chloride (Biopolymer, USA), M-Endo broth medium, Difco, (USA), M-FC broth medium, Difco, (USA), nutrient agar, Difco, (USA), sodium fluorescein, Aldrich, (Puriss), chromium chloride (CrCl_2_), ferric chloride (FeCl_3_), lead acetate Pb(CH_3_COO)_2_), copper sulphate (CuSO_4_) and cadmium sulphate (CdSO_4_), Aldrich, (Germany), nitric acid (HNO_3_), Merck, (Germany) and distilled water (Nanopure Produced by Millipore instrument).

### 2.2 Methods

#### 2.2.1 Extraction of chitosan from the crabs shell wastes

Atypical method to extract chitosan from the crab's shells wastes was obtained from the local markets and suspended in 4 % HCl (Puriss, Fluka) at room temperature in the ratio of 1:14 (w/v) up to 36 hrs and the residual was washed with water to remove the acid. The deproteinization of shells was done by treating the residual with 5 % NaOH (Puriss, Fluka) at 90 °C for 24 hrs with a solvent to solid ratio of 12:1 (v/w). After the incubation time, the shells were washed to neutrality in running tap water and sun-dried, the product obtained was chitin.

Chitosan preparation involves the deacetylation of the obtained chitin (Dutta et al., 2004). By employing 70 % NaOH (Puriss) solution with a solid to liquid ratio of 1:14 (w/v) and incubated at room temperature for 72 hrs. The residue obtained after 72 hrs. washed with running tap water to neutrality and rinsed with deionized water. The product was sun-dried and finely grinded to the powder chitosan.

#### 2.2.2 Preparation of silver nanoparticles (Ag-NPs) via chemical reduction method

Silver nitrate (AgNO_3_, BDH), Chitosan and trisodium citrate (C_6_H_5_O_7_Na_3_) of analytical grade were used as starting materials without further purification. Silver nanoparticles were prepared by using a chemical reduction method as shown in Eq. (1). In a typical experiment 10 ml of 10^−2^ mol dm^−3^, AgNO_3_ was added to 250ml of distilled water and heated to boiling then 2 gm of chitosan was added, then 10 ml of 1 % trisodium citrate was added dropwise. The solution was heated until a color change is evident (pale yellow). Then the heating stopped and continue stirring until cooled to room temperature then the stocked sample contains 2 gm chitosan, with the actual concentration of 4x10^−4^ mol dm^−3^ of Ag-NPs.

(1) 4Ag+ + C_6_H_5_O_7_Na_3_ + 2H_2_O → 4Ag^0^ + C_6_H_5_O_7_H_3_ + 3Na+ + H^+^ + O_2_

#### 2.2.3 Preparation of thin films

The thin films of CS/Ag bionanocomposites were prepared within five different thin films according to the following method. In the first, thin film 100 ml of 1% of Poly (vinylidene chloride) (PVDC) biopolymer was used without silver and chitosan as a control. The other four films were prepared by mixing 20, 40, 60, 80 ml of the stock solution of the nanocomposites and completed to 100 ml by1% of biopolymer to give (0.16%, 8x10^−5^ mol dm^−3^), (0.32%,1.6x10^−4^ mol dm^−3^), (0.48%,2.4x10^−4^ mol dm^−3^), (0.64%,3.2x10^−4^ mol dm^−3^), respectively. The mixtures were poured in a plastic box of (20 × 30 cm) diameter and drying at ambient temperature.

#### 2.2.4 Preparation of agar for microbial activity

The antimicrobial activity of silver nanoparticles was studied by (Colony Forming Unit) CFU/cm^2^ measurements against tow pathogenic bacterial Strains, gram-negative bacteria like *E. coli*, and gram-positive bacteria like *G. bacillus,* By using different concentrations of chitosan and Ag-NPs at 4x10^−4^ mol dm^−3^ and 0.8 % chitosan. The agar media with and without chitosan and Ag-NPs were prepared for the same culturing strains. Both were autoclaved and poured into different Petri dishes.

#### 2.2.5 Preparation of MFC agar for aerobic fecal coliforms bacteria for colony count

In a classic experiment, 52 gm of the MFC powder was suspended in 1 L of double distilled water and heated with frequent agitation and boiled for 1 min to completely dissolve. Then 10 ml of 1% solution of rosolic acid in 0.2 mol dm^−3^ NaOH was added and continued heating to another 1 min. The mixture was adjusted to pH 7.4 with dilute HCl. A typical control culture used without different concentrations of mixture with chitosan and Ag-NPs 4x10^−4^ mol dm^−3^and 0.8% chitosan was prepared and incubates at temperature 44.5 °C for 48 hr.

#### 2.2.6 Preparation of M-Endo agar for aerobic total coli forms bacteria for colony count

In this case 51 gm of the M-Endo powder was added to 1 L l of double distilled water containing 20 ml of 95 % ethanol. The mixture was heated with stirring and boiled for 1minute to completely dissolving the powder. A characteristic control cultures used without different concentrations of the mixture with chitosan and Ag-NPs 4x10^−4^ mol and 0.8 % chitosan was prepared and incubate at temperature at 35 °C for 24 hrs.

#### 2.2.7 Preparation of nutrient agar for inhibition zone method

In 1 L of double distilled water, 23 gm of the nutrient agar was added and then boiled to 1 min to completely dissolving. The experiment was repeated by using different concentrations of the mixture of chitosan and silver nanoparticles for monitoring of the microbial activity by inhibition zone method and incubates at 37 °C for 24 hrs. The cultures without chitosan and nanoparticles were kept as a control for a comparison study. Then two bacterial strains (*E. coli* and *G. bacillus)* were grown in nutrient broth medium and were screened in the lowest concentration of an antimicrobial agent which will prevent the growth of microorganisms after overnight incubation.

### 2.3 Characterization

UV-visible absorbance spectra of prepared samples were measured using *JENWAY 6800* double beam spectrophotometer. FT-IR spectrometer (Germany) with range (5000-370 cm^−1^). Size and shape measurements were carried out by a transmission electron microscope (TEM), JEOL (JEM-1400). X-ray diffraction measurements were carried out using a Philips X'Pert-Pro Powder Diffract meter operating with a Cu anode with Kα_1_ = 1.54060Ǻ, Kα_2_ = 1.5444 Ǻ. Analysis of heavy metals by using Inductively Coupled Plasma Optical Emission Spectrometry [ICP- OES], Varian liberty series II, Italy. The air flow experiments of the aqueous sample were carried out by using a peristaltic pump variable flow (AQUARIUM AIR PUMP) (CHINA) Model: (CA-8200).

#### 2.3.1 Adsorption experiment

In a plate's box containing CS/Ag bionanocomposites thin films, 1000 ml of 200 ppm of each heavy metal (Cd, Pb, Cr, and Fe) solution were added. The control experiment was carried out with different percent of chitosan thin films. The samples were preserved immediately after collection via acidification by adding 5 ml nitric acid to each sample. After acidification, the samples were taken out at regular time intervals of 30 mins and then analyzed by using (ICP-OES).

#### 2.3.2 Photocatalysis experiments by using natural sunlight

In a Plat containing the polymer thin film, 500 ml of the dye was added. The solution was then illuminated in the natural sunlight. The integrated light intensity between 300-800nm was in the range 280–600 W/m2. The concentration of the dye was 2x10-5 mol dm-3 and for sodium fluorescein. Two control experiments were carried out, one in dark in the presence of a different concentration of thin films prepared and the other one exposed to light. Samples were taken out at regular time intervals 30 min and analyzed by using jenway6800 double beam spectrophotometer. The experiment was repeated in light in the presence of a different concentration of thin films prepared of silver nanoparticles coated with the polymer and polymer used as it is.

## 3 Results and discussion

### 3.1 UV-visible absorption spectra of CS/Ag bionanocomposites

**Fig. 1:**
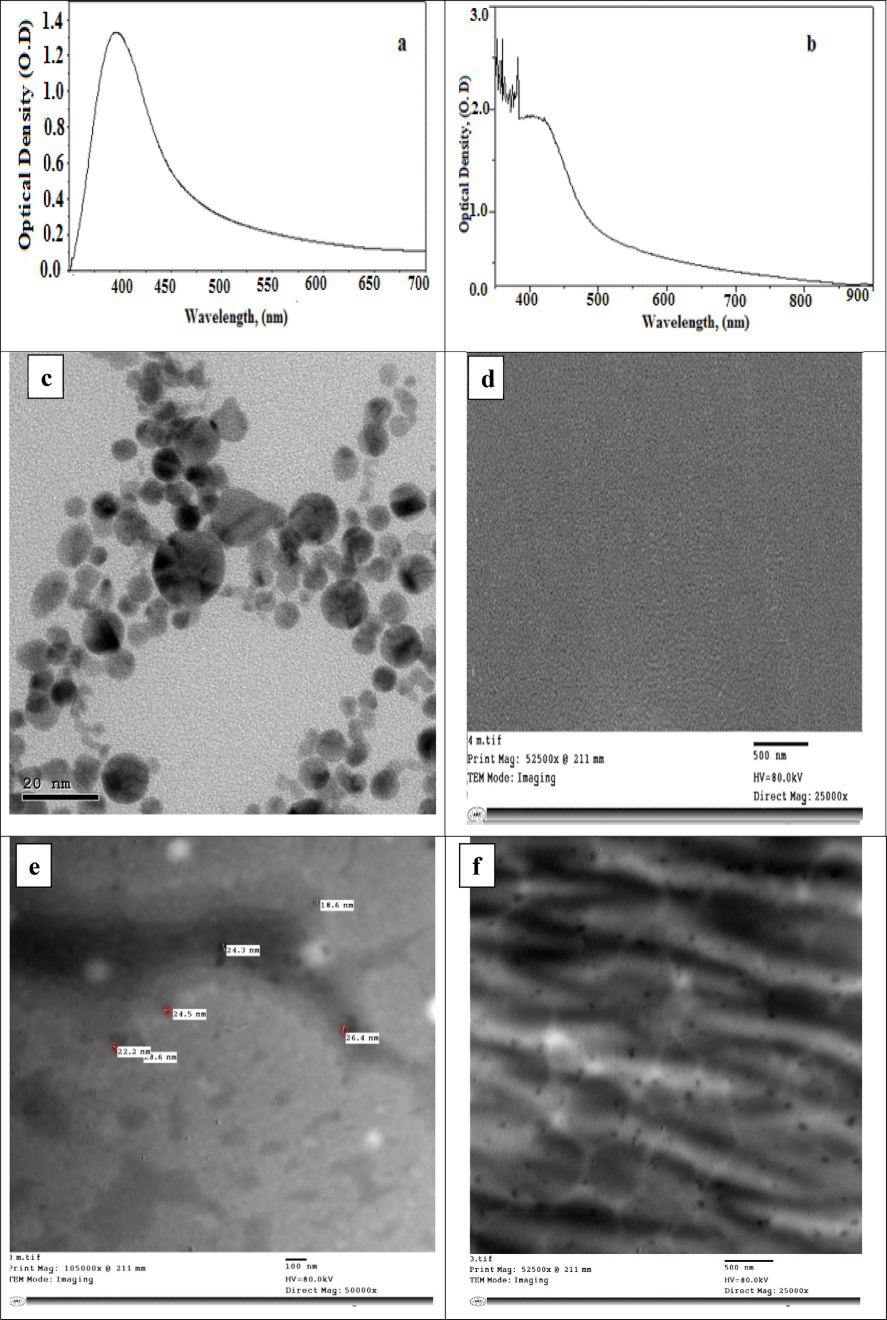
a) UV-Visible absorption spectra of Ag-NPs, b) UV-Visible absorption spectra of CS/Ag bionanocomposites, c) Transmission electronic microscope (TEM) of Ag-NPs, d) TEM of CS/PVDC thin film, e) TEM of CS/Ag nanocomposites, and f) CS/PVDC/Ag bionanocomposites.

UV-vis spectroscopy performance is comprehensively used to examine the optical properties of the synthesized nanoparticles as well as the prepared bionanocomposites. Fig. (1a) illustrates that, UV–vis spectra of silver nanoparticles which showing only one Plasmon absorption band at λ_max_ = 398 nm. The spectrum indicated that the prepared silver nanoparticles have the spherical shape. In the case of CS/Ag bionanocomposites (Fig. 1b), it was displayed that, the absorption spectra was at λ_max_ = 412 nm with a detectable broad band observed at a longer wavelength of the visible region extended to 900 nm. The spectrum indicated that the addition of chitosan has a slight effect on silver nanoparticles with a small increase in size without changing in the shape. The detectable broadening absorption band might be due to the light scattering of some aggregated of CS/Ag bionanocomposites.

### 3.2 Morphological studies of CS/Ag nanocomposites

To get the confirmation of the formation of silver nanoparticles coated by chitosan and silver nanoparticles coated by chitosan and PVDC as biopolymer, high-resolution transmission electron microscopy (TEM) analysis is performed.

Fig. 1 (c, d) represents the TEM micrograph of Ag-NPs which appear in nanoscale from 5-15 nm in homogeneous dispersion, also the thin film fabricated from chitosan and biopolymer (CS/PVDC) seem as very uniform and more compatible between CS and PVDC as shown in (Fig. 1 d). Furthermore, (Fig. 1 e), revealed that the loaded Ag-NPs on the chitosan matrix are achieved to be nanosized range about ∼20 nm. Correspondingly, the (Fig. 1 f) demonstrated that the presence of silver nanoparticles as highly dispersed black dots over and in-between the thin film of CS/PVDC/Ag bionanocomposites. From all figures, it can be observed that the shape of silver nanoparticles is a spherical shape with no changes in the morphology or the shape with adding of chitosan or the biopolymer.

### 3.3 FT-IR spectroscopic characterization of PVDC/CS/Ag bionanocomposites

**Fig. 2:**
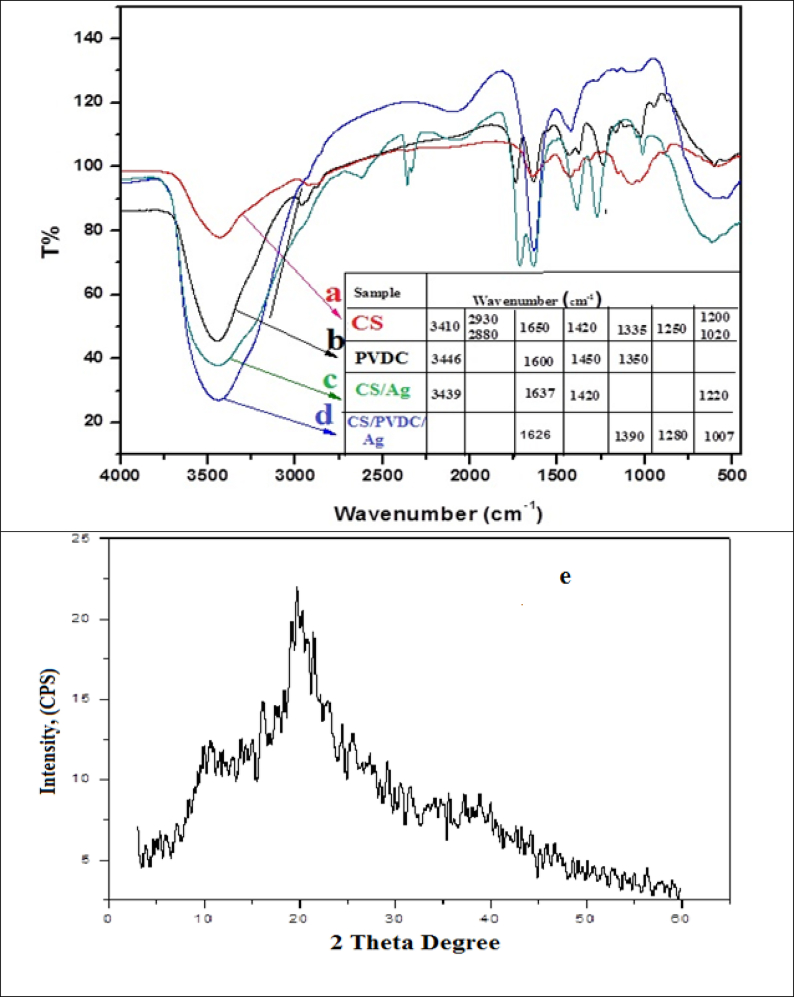
FT-IR spectra of a) Chitosan, b) PVDC, c) CS/Ag nanocomposites, d) CS/PVDC/Ag nanocomposites, Fig. 2e: XRD pattern of chitosan prepared from shrimp processing waste (shells).

From Fig. 2 (a, b, c and d) which reveals the FT-IR characterization of CS, PVDC, CS/Ag bionanocomposites as well as PVDC/CS/Ag bionanocomposites. In the case of chitosan, the major absorption band is observed between 1220 and 1020 cm^−1^ which represents the free amino group (-NH_2_) at the C_2_ position of glucosamine, a major group present in chitosan. Further to the study, Fig. (2a) showed another absorption band at 1335 cm^−1^ characterizes the -C-O stretching of the primary alcoholic group (–CH_2_– OH). The absorbance bands at about 3410, 2930, 2880, 1650, and 1420 cm^−1^ indicated the N–H stretching, symmetric CH_3_, and asymmetric CH_2_, CH stretching, C=O stretching in secondary amide (amide I) and C–N- stretching in secondary amide (amide II), respectively. This confirms the structure of chitosan.

In this study, the PVDC biopolymer has been used to increase the strength of the thin films. Fig. (2b) indicated that the FT-IR has strong absorption around 1600 cm^−1^ which attributed to C=C stretching. The phenyl ring vibrations are assigned to absorption near 1600 and 1500 cm^−1^. The CH_3_ asymmetric and symmetric bending modes are traced to 1450 and 1350 cm^−1^, respectively. The CH_2_ bending mode also appears around 1455 cm^−1^. In the case of CS/Ag bionanocomposites Fig. (2) displayed that the red shift of the spectra from 1020 cm^−1^ to 1220 cm^−1^ and disappearance at 2880 cm^−1^. The red shift observed illustrated that Ag loaded in chitosan films.

Moreover, the absorbance bands of 3439 cm^−1^, 1637 cm^−1^ and 521 cm^−1^, which indicated the presence of molecular functional groups that are responsible for the reduction of silver ions as shown in (Fig. 2b), these functional groups may be used as capping agents and stabilizing the silver ions. Though the exact mechanism involved in the formation of Ag-NPs nanoparticles is still debated. Fig. (2d) demonstrates the FT-IR spectra of the mixture of chitosan, Ag nanoparticles and poly (vinylidene chloride) PVDC.

From Fig. 2d it can be observed that the blue shifts of the absorption bands at 1025 cm^−1^ and 1650 cm^−1^ to 1007 cm^−1^ and 1626 cm^−1^, which is corresponding to the CO stretching, this indicating that the binding of the CS/Ag bionanocomposites to the PVDC. The CH_3_ symmetric bending modes located at 1350 cm^−1^ are shifted to 1390 cm^−1^, this clearly demonstrating that the silver binds to the functional groups of chitosan. Peak shifting occurs due to coordination between the metal atom (silver in this case) and electron rich groups (oxygen/nitrogen).

### 3.4 X-ray diffraction (XRD)

XRD patterns chitosan are illustrated in (Fig. 2e). The XRD pattern of chitosan exhibits broad diffraction peaks at 2θ = 10° ±1 and 21° ±1 which are typical fingerprints of semi-crystalline chitosan (Bangyekan et al., 2006). Yen and Mau (2007) founded that chitosan showed two crystalline reflections at 9.7° and 19.9°. Prashanth et al. (2002) were established that the XRD patterns of shrimp chitosan showed two major characteristic peaks at 2θ = 9.9–10.7° and 19.8–20.7°. It is also reported that the two characteristic crystalline peaks with slightly fluctuated diffraction angles found in the XRD patterns indicated that two types of α- and γ-chitosan exhibited a comparable degree of crystallinity and had two consistent peaks of 9–10° and 19–20°. Also, Chitosan confirmed that it can be used commercially in different fields such as food supplement, additive, drug preparation as well as water treatment. The preparation of chitosan from shrimp processing waste (shells) would successfully minimize the environmental pollutants.

### 3.5 Photodegradation of organic pollutants on CS/PVDC/Ag bionanocomposites

**Fig. 3:**
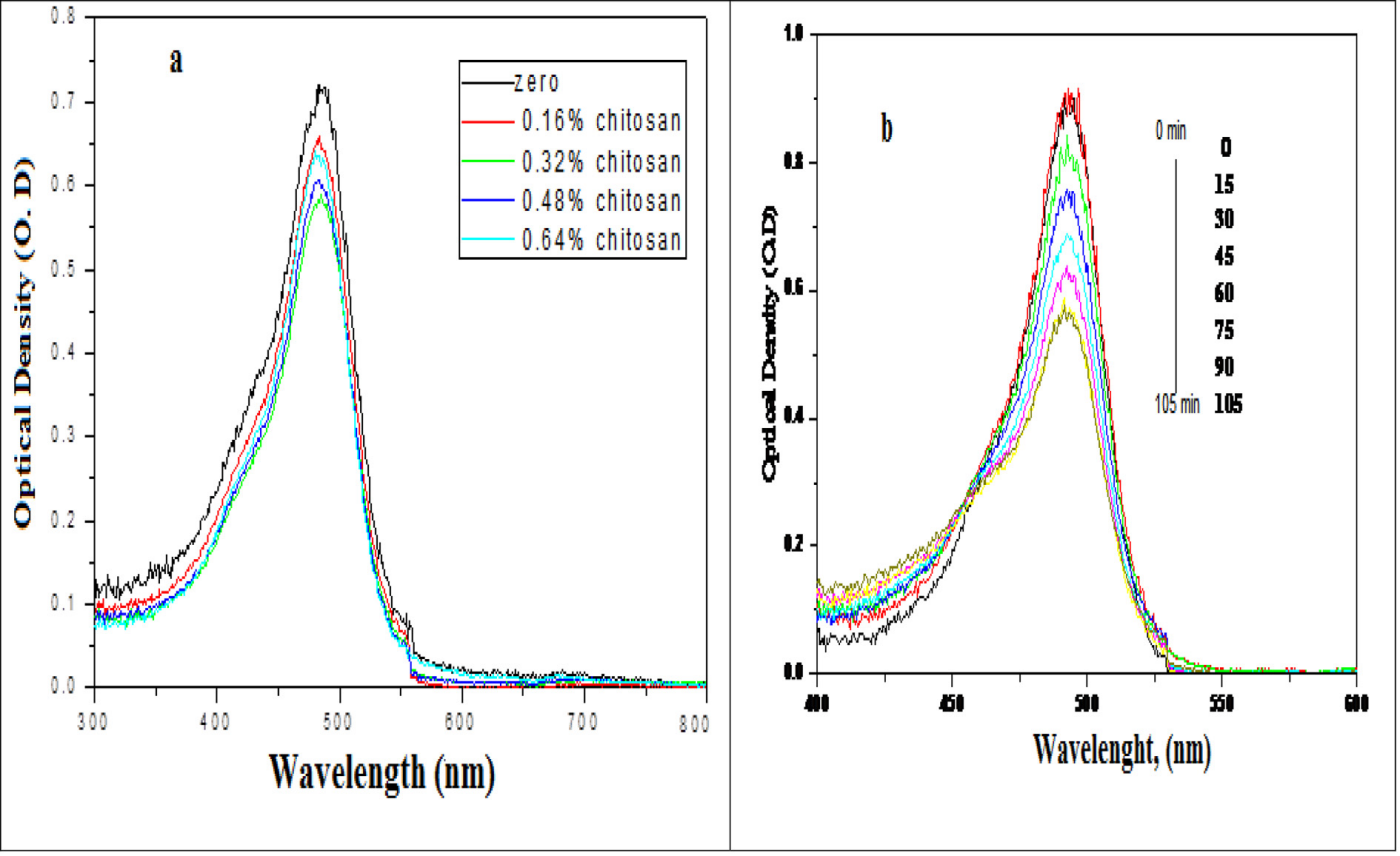
Absorption spectra changes of the adsorption of 2x10^−5^ mol dm^−3^ of sodium fluorescein in (a) sunlight without thin film and (b) dark over CS/Ag nanocomposites thin films at the ratios of (0.16 %, 0.32%, 0.48%, and 0.64%).

**Fig. 4:**
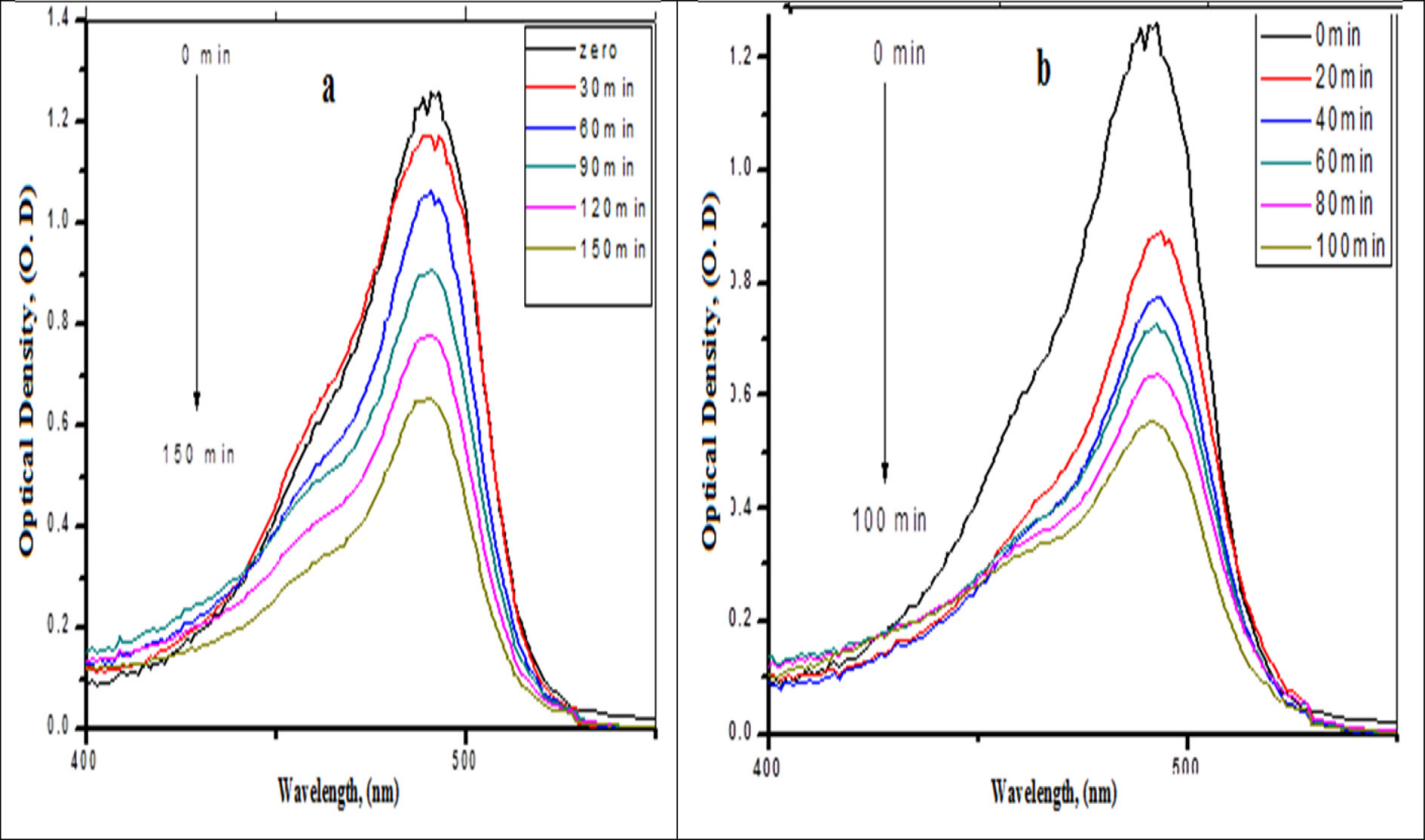
(a, b): Absorption spectra changes of photodegradation of 2x10^−5^ mol of sodium fluorescein dye under sun light as a function of the irradiation time, (a) Sunlight without aeration and (b) Sunlight with aeration on 0.48% chitosan and 2.4x10^−5^ mol dm^−3^of Ag-NPs.

One of the most important criteria for an efficient charge transfer is to adsorb the organic pollutant strongly on the CS/Ag bionanocomposites thin films surfaces. In other words, pre-adsorption of the organic pollutant is very important. Sodium Fluorescein dye was used as a model of organic pollutants. 500 ml of 2x10^−5^ mol dm^−3^ of the dye was used at different concentrations of chitosan and silver nanocomposites thin films. Fig. (4.a and b) Shows slightly degradation of the dye in the sunlight after ∼2 hours without thin film about 30% of the dye was degraded, also slightly decreasing in the optical density, ∼10%, 12%, 16%, 19%, is observed in the dark after 1 hr. in the presence of the thin films. This attributed to the adhesion of sodium fluorescein. This slightly decreasing in the optical density became sufficient to the photo-degradation of the dye on the thin films. From Fig. (3 a, b) the desired process was at 0.48% of chitosan thin film.

### 3.6 Photodegradation under sun light without and with aeration

Due to the higher photocatalytic activity under UV-Visible light and accordingly the charge transfer between excited sodium fluorescein molecules and the system components were more efficient on the surface especially in the presence of Lon pair of electrons of the amino group of chitosan. Also, the presence of the plasmonic band of silver nanoparticles (λ = 412 nm) and an intense absorption band of Sodium Fluorescein in the visible region (λ = 487 nm) may enhance the photocatalytic activity of UV-Visible light. The time courses of the absorption spectra during the photodegradation process of the dye under sun irradiation at 0.48% of chitosan thin film without and with aeration are illustrated in Fig. 4 (a, b). As observed in the Figure, after irradiation to 150 mines about 48 % of the dye was degraded as shown in Fig. 4a, while in the presence of aeration ∼75% of the dye was degraded after 100 min.

The normalized absorption spectrum of sodium fluorescein dye at the irradiation time courses under the sunlight is shown in Fig. 4 b which showed a slight adsorption of the dye on the control of the polymer thin films at different concentrations of chitosan and silver nanoparticles ∼10%, 12%, 16%, 19% for 0.16%, 0.32%, 0.48%, 0.64%, respectively, were adsorbed. This Figure also indicated the photo-degradation of sodium fluorescein decolorization on polymer/chitosan/silver nanoparticles thin films at different ratios in the presence and absence of oxygen. After 2.5 hrs about 48% of the dye without aeration was photo-degraded in the presence of the polymer thin films with the photodegradation rate of about 7.26x10^−5^ mol dm^−3^ Sec.^−1^ (Honary et al., 2011). While at the same time with the aeration the photodegradation was 51% with the degradation rate about 1.36x10^−4^ mol dm^−3^ Sec.^−1^ (Honary et al., 2011).

In case of the CS/PVDC/Ag bionanocomposites thin films at the same time about 51%, 58%, 61% and 63% of the dye without aeration was photo-degraded at different ratios of 0.16 %, 0.32%, 0.48%, 0.64% and the photodegradation rates were 8.10x10^−5^, 9.90x10^−5^, 1.03x10^−4^ and 1.13x10^−4^ mol dm^−3^ Sec.^−1^, respectively. While the photodegradation monitored about 64%, 69%, 72% and 77% at the same ratios of chitosan with aeration and the photo-degradation rates calculated were 1.74x10^−4^, 2.12x10^−4^, 2.18x10^−4^ and 2.37x10^−4^ mol dm^−3^Sec.^−1^ by the following equation:

Ln C_0_/C_f_ = KT

Where C_0_ is initial concentration mole (dm^−3^) and C_f_ is final concentration mole (dm^−3^) and T time/second and K The rate of photodegradation of sodium fluorescein dye.

**Fig. 5:**
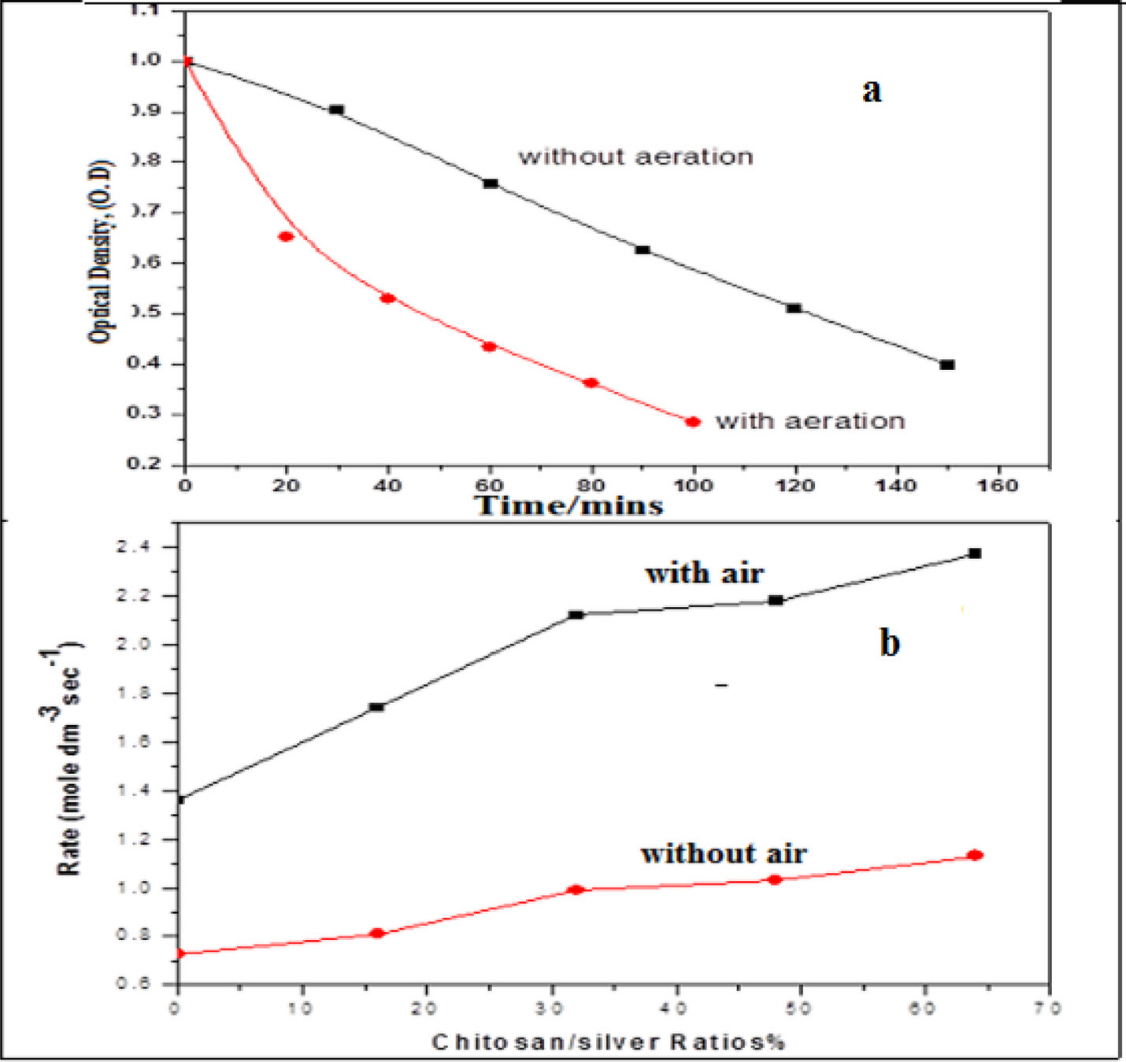
a) Dependence of the normalized concentration of sodium fluorescein dye using CS/PVDC/Ag bionanocomposites thin films coated on polymer with and without air under irradiation, b) Rates of photodegradation at different ratios of CS/PVDC/Ag bionanocomposites thin films.

The rates of photodecomposition at the time courses of CS/Ag bionanocomposites polymer were summarized in Table (1) and Fig. (5a), and shows that it is approximately faster than the one catalyzed by chitosan/polymer beads. The table illustrated that the presence of CS/Ag bionanocomposites was efficient in photodegradation and the rat in case of aeration is faster than in the absence of aeration. Since the position of the absorption peak of sodium fluorescein dye at λ = 487 nm was not changed through the adsorption on the polymer beads as well as no photodegradation observed of the dye in the dark only during exposure to the sunlight. Also no changes in the absorption position during the photodegradation processes, the mechanism may depend upon both the dye and the CS/Ag nanocomposites

**Table 1:** Summarizes the photodegradation rates on the polymer nanocomposite with and without air.

Thin films	Ag-NPs, %	The Rate With air mole dm^−3^ Sec.^−1^	The rate Without air mole dm^−3^ Sec.^−1^
CS/PVDC/Ag nanocomposite	0.16	1.74x10^−4^	8.10x10^−5^
CS/PVDC/Ag nanocomposite	0.32	2.12x10^−4^	9.90x10^−5^
CS/PVDC/Ag nanocomposite	0.48	2.18x10^−4^	1.03x10^−4^
CS/PVDC/Ag nanocomposite	0.64	2.37x10^−4^	1.13x10^−4^

Then the redox reactions are initiated from the excitation of CS/Ag bionanocomposites as well as sodium fluorescein. In this context, most of the visible light with λ < 400 nm (1) illuminations only excites sodium fluorescein and the excited molecules can inject electrons on to the surface of chitosan/silver polymer beads. On the other side, the photocatalytic properties of Ag nanoparticles in visible light may well be due to excitation of surface plasmon resonance (SPR), which is nothing but the oscillation of charge density that can propagate at the interface between Ag and dielectric medium_._ Wang et al. (2009) stated that the Ag-NPs are good, highly efficient and stable photocatalysis under ambient temperature with visible light illumination for degrading organic compounds and dyes. So, at the surface of the chitosan/silver nanocomposites the photogenerated electron (e^−^) is produced as a result of the absorption of a photon of higher energy, λ < 400 from the amino group of the chitosan leaving positive charge on the nitrogen atom and the positive charge generated oxidizes sodium fluorescein adsorbed after diffusion to the surface from the trapped photogenerated Cs/Ag OH^.+^ (Yen et al., 2009) all of the injected electrons react with the surrounding oxygen molecules occurs to yields superoxide (2) and other radicals of HO^.2^, HO^−2^, H_2_O^2^, ·OH which play a major role in the photocatalysis of sodium fluorescein dye. In the presence of silver nanoparticles, it has been shown that the photocatalytic electron transfer process can be enhanced by depositing on the silver nanoparticles at the chitosan interface.

As shown in Fig (5b) the photodegradation rate of sodium fluorescein dye was 7.26x10^−5^ mol dm^−3^ Sec^.−1^ incase polymer beads without air while the photodegradation rates under the same condition in the presence of CS/Ag bionanocomposites at different ratios of 0.16 %, 0.32%, 0.48%, 0.64%, the photodegradation rates were 8.10x10^−5^, 9.90x10^−5^, 1.03x10^−4^ and 1.13x10^−4^ mol dm^−3^ Sec^−1^, respectively. In the presence of oxygen, the photodegradation rate was 1.36x10^−4^ mol dm^−3^ Sec^.−1^, for polymer bionanocomposites, while it was in CS/Ag bionanocomposites at different ratios of 0.16 %, 0.32%, 0.48%, 0.64% with aeration the photodegradation rates were 1.74x10^−4^, 2.12x10^−4^, 2.18x10^−4^ and 2.37x10^−4^ mol dm^−3^ Sec^.−1^, respectively. This means that the presence of CS/PVDC/Ag bionanocomposites beads enhances the rate of photodegradation more than that of polymer beads and increases with increasing the ratios.

### 3.7 Efficiency of thin film in removing heavy metals

Batch adsorption experiments of heavy metals of such as iron, cadmium, lead, and copper were carried out to determine the adsorption capacity of chitosan/silver/polymer nanoparticles thin films with ratios 0.16%, 0.32%, 0.48%, 0.64% of CS and PVDC biopolymer used without nanoparticles as control and the concentrations of heavy metals will be started at 200 ppm to calculate the adsorption rates. Adsorption experiments carried out by added 1000 ml of 200 ppm in the plates containing the thin films at pH ranging from (5.5–6.5) and temperature 25 °C. The metals concentrations including, iron, cadmium, lead, and copper will be measured at λ = 259.940 nm, 226.502 nm, 220.353 nm, 324.754 nm, respectively, by using inductively coupled plasma (ICP-OES).

**Fig. 6:**
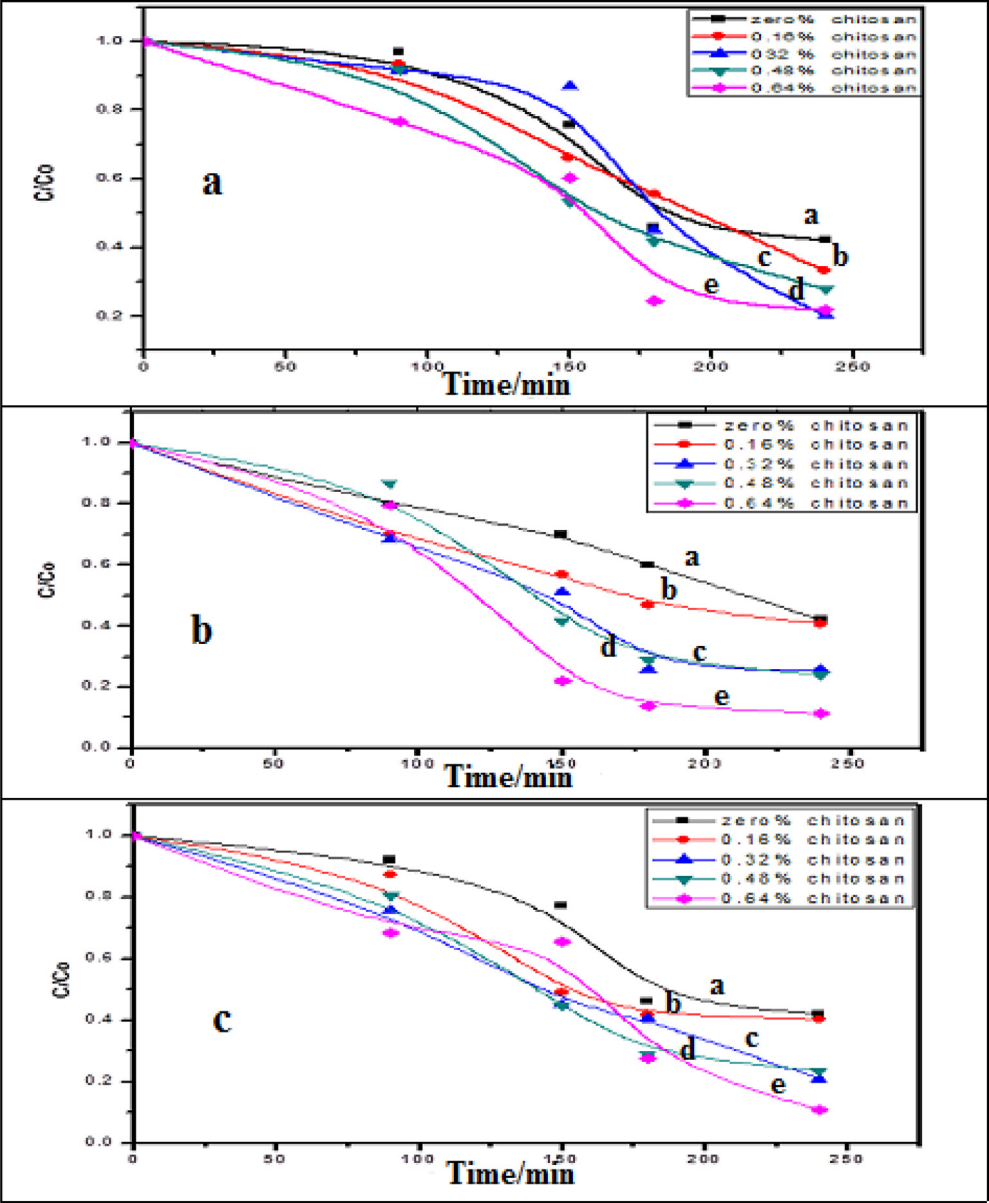
The rate of removal, a) Cu, b) Pb, c) Cd at different concentrations of CS/Ag nanocomposites, a) PVDC, b) 0.16% chitosan and 8x10^−5^ mol dm^−3^of Ag, c) 0.32% chitosan and 1.6x10^−4^ mol dm^−3^of Ag d) 0.48% chitosan, 2.4x10^−4^ mol dm^−3^of Ag and e) 0.64% chitosan and 3.2x10^−4^ mol dm^−3^ of Ag-NPs.

Adsorption of cupper carried out by using different ratios of 0.16%, 0.32%, 0.48%, and 0.64% of CS/Ag bionanocomposites thin films as adsorbents. The cross-linked were synthesized by the homogeneous reaction of chitosan/silver/polymer thin films in an aqueous acetic acid solution that had been used to investigate the adsorptions of copper metals Cu (II) ions in aqueous solution. Fig. 6a displays the normalized curves of the adsorption of copper metals Cu (II) ions in aqueous solutions at the mentioned ratios above which indicated that the percent removal were 67%,71%,77%, and 97% with the rates of adsorption of 7.5 x10 ^−5^ mol dm^-3^ s^-1^, 8.7x10 ^−5^ mol dm^-3^ s^-1^, 1.0 x10 ^−4^ mol dm^-3^ s^-1^, 1.10 x10 ^−4^ mol dm^-3^ s^-1^,respectively. From the curve, we can note that the increases in the ratios of chitosan polymer accompanied the increasing of the adsorption of Cu metal ions.

Adsorption of lead ions carried out using verity ratios 0.16 %, 0.32%, 0.48%, 0.64% of CS/Ag bionanocomposites thin films as adsorbents. Fig. 6b offered the normalized curves of the adsorption of Pb metal ions at the same different ratios indicates that the percent removal was 59%,73%,75%, and 88% with the rates of adsorption of 6.3 x10^−5^ mol dm^-3^ s^-1^,9.0 x10^−5^ mol dm^-3^ s^-1^,9.7 x10^−9^ mol dm^-3^ s^-1^,1.4 x10^−4^ mol dm^-3^ s^-1^, respectively. From the curve, we can note that the increases in the ratios of chitosan polymer accompanied the increasing of the adsorption of Pb metal ions.

Fig. 6c displayed that the normalized curves of the adsorption of Cd ions at different ratios of 0.16 %, 0.32%, 0.48%, and 0.64% of CS/Ag bionanocomposites thin films as adsorbents. Fig. 6c indicated that the percentage of removal of Cd metal ions are 59%, 76%, 78%, and 89% with the rates of adsorption of 6.2 x10^−5^ mol dm^-3^ s^-1^, 9.9 x10^−5^ mol dm^-3^ s^-1^, 1.06 x10^−4^ mol dm^-3^ s^-1^, 1.5 x10^−4^ mol dm^-3^ s^-1^, respectively. From the curve, we can note that the increases in the ratios of chitosan polymer accompanied the increasing of the adsorption removal of Cd metal ions.

### 3.8 Antimicrobial activity

#### 3.8.1 Colony count assay

Quantitative measurement of microbial activity of silver nanoparticles and CS/Ag bionanocomposites were performed against two different bacterial strains like *E. coli* with bacterial count 440 colony forming unit CFU/cm^2^ and *G. Bacillus* with bacterial count 26000 CFU/cm^2^ were taken as a model of bacteria strains. The mixture of silver was prepared with a concentration of 4x10^−4^ mol dm^−3^ and the mixture of CS/Ag were 4x10^−4^ mol dm^−3^ silver and 0.8% chitosan by preparing different ratios as (1:2.5, 1:5, 1:10, 1:20, 1:40, 1:60, 1:80 and 1:100), and grown on specific media like M-Endo and MFC agar and incubate at temperature at 35 °C for 24 hrs, and 44.5 °C for 48 hrs, respectively.

Our results have revealed that CS/Ag bionanocomposites clearly show greater antimicrobial activity effects than silver nanoparticles. The microbial colony count of bacteria was estimated by using colony count assay for silver and CS/Ag were summarized in (Table 2).

**Table 2:** Bacterial growth count of *E. Coli and G. Bacillus.*

Concentration (mole dm^−3^)	Bacterial growth counts (CFU/cm^2^)			
	*G. Bacillus* Ag-NPs	*E. coli* Ag-NPs	*G. Bacillus* CS/Ag	*E. coli* CS/Ag
4x10^−6^	25600	405	24800	310
5x10^−6^	21400	335	22000	240
6.4x10^−6^	19200	310	20200	75.0
1x10^−5^	19600	275	18000	16.0
2x10^−5^	13200	255	12800	7.00
4x10^−5^	3100	205	2900	0.00
8x10^−5^	370	110	210	0.00
1.6x10^−4^	0.00	0.00	0.00	0.00

**Fig. 7:**
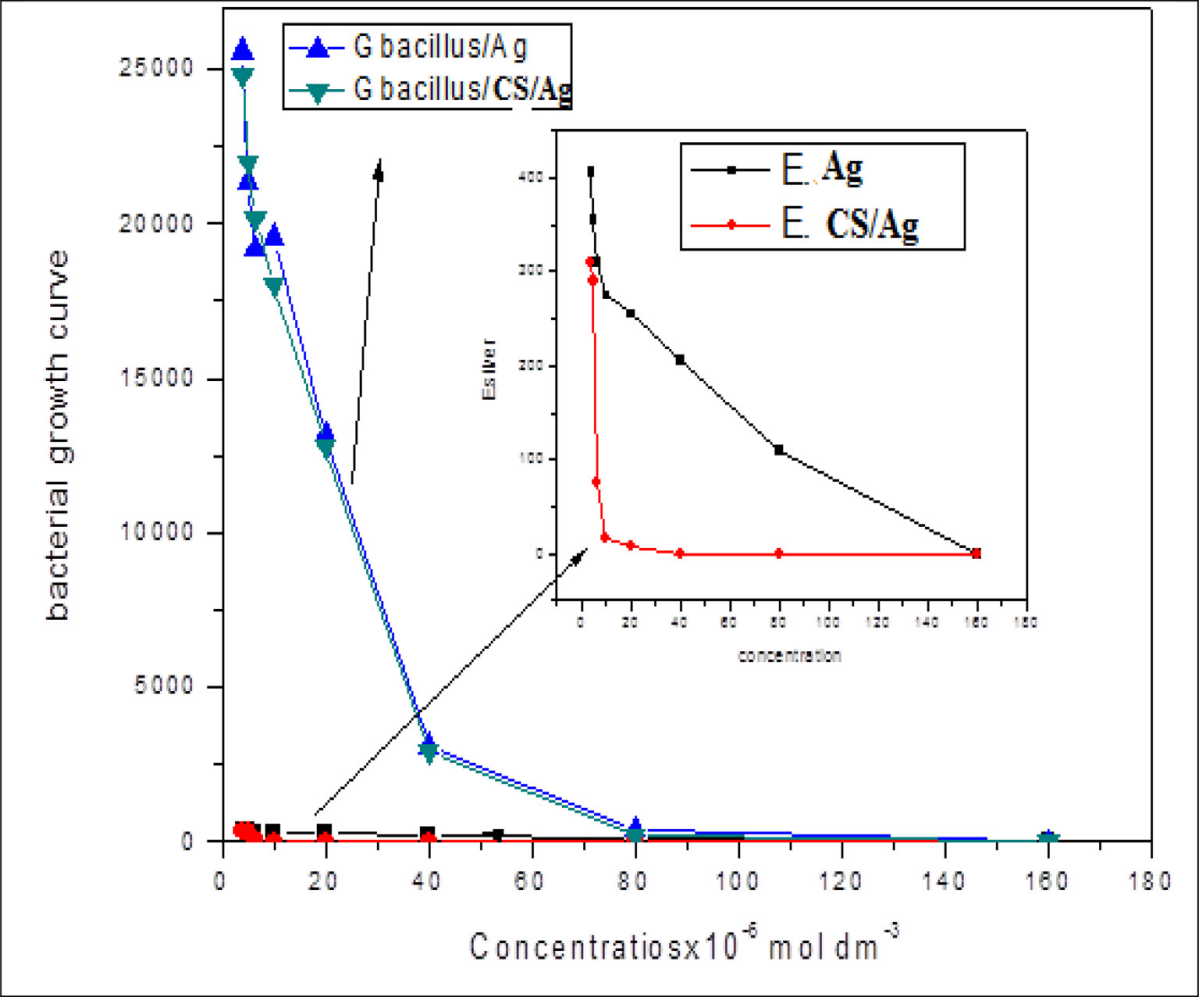
Shows types of bacterial growth curve of a) *E. coli* and, b) *G. bacillus* against different concentrations of Ag-NPs and CS/Ag nanocomposites.

In the present study, the antimicrobial activity of CS/Ag bionanocomposites displayed antibacterial activity toward the gram-negative bacteria *(E. coli)* with zero bacterial counts than the gram-positive bacteria *(G. bacillus)* with zero bacterial count, so it can be concluded that the effect of antibacterial activity is higher by using CS/Ag bionanocomposites rather than silver nanoparticles that shown in (Fig. 7).

#### 3.8.2 Inhibition zone method

Qualitative measurement of microbial activity carried out by the same bacterial strains and different concentrations of silver nanoparticles and CS/Ag bionanocomposites. The same two bacterial strains were grown on nutrient agar. The inhibitory activity was measured based on the diameter of the clear inhibition zone after incubation at 37 °C for 24 hrs. Our results have revealed that CS/Ag bionanocomposites display a greater inhibitory effect than silver nanoparticles.

**Fig. 8:**
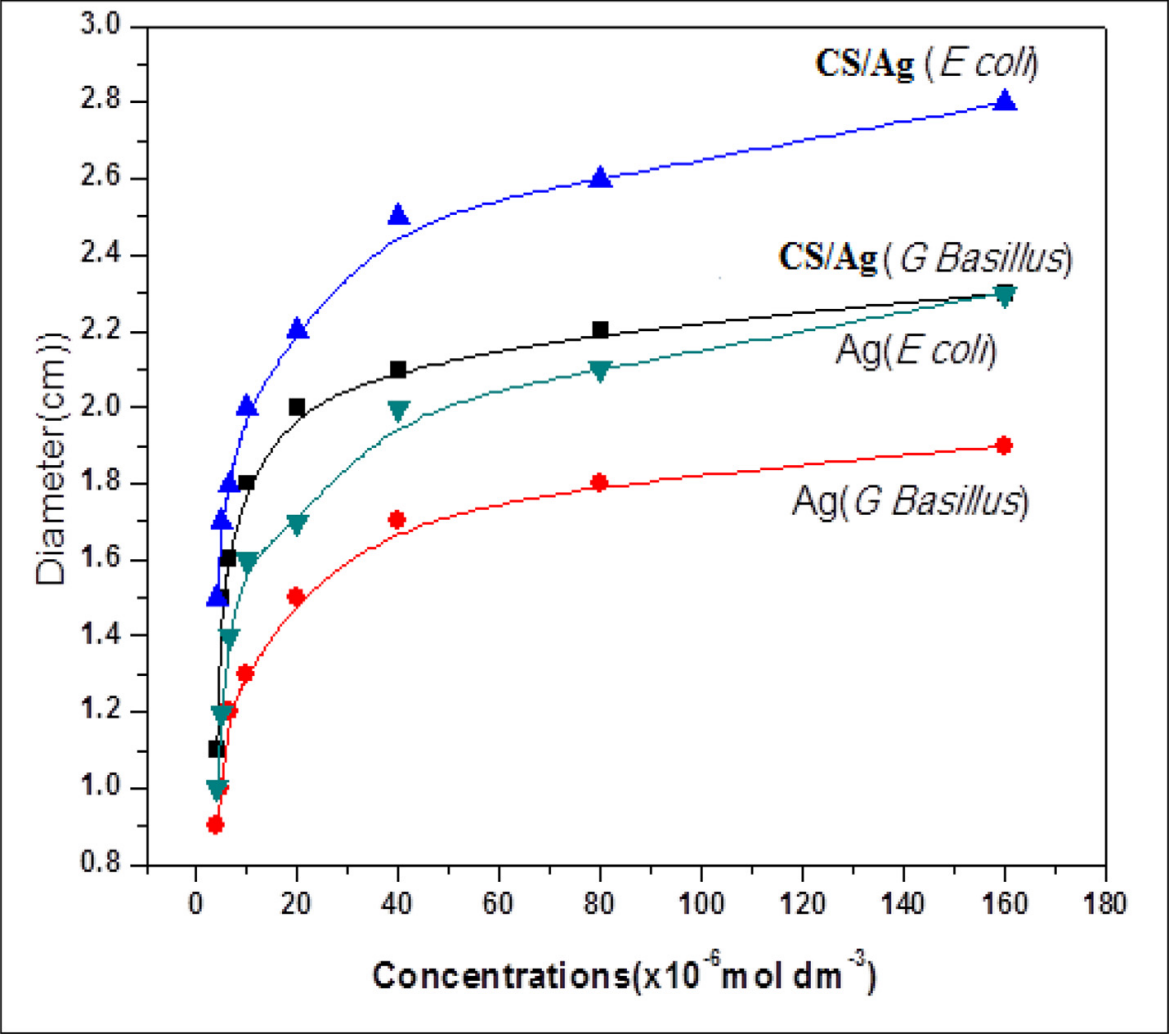
Shows types of bacterial strains inhibition zone of *E. coli* and *G. bacillus* against different concentrations of Ag-NPs and CS/Ag bionanocomposites.

In the present study, the antimicrobial activity of CS/Ag bionanocomposites revealed good antibacterial activity toward gram-negative bacteria *(E. coli)* with maximum inhibition zone around (2.8) cm than gram-positive bacteria *(G. bacillus)* with inhibition zone around (2.2) cm. owing to the release of diffusible of silver nanoparticles used based on Fig. (8), so it can be concluded that the effect of antibacterial activity is higher by using CS/Ag bionanocomposites rather than silver nanoparticles that are shown in (Fig. 8).

## 4 Conclusions

Designing of chitosan and thin films of PVDC coated with Ag-NPs (CS/PVDC/Ag) is a successful technique for the photodegradation of organic pollutants under sunlight irradiation with and without oxygen. The photodegradation rate of the dye was found to be increased with raising catalyst concentrations using incorporated polymer thin films. It is important to mention that chitosan and Ag-NPs pounded to the polymer thin film is a successful technique for the catalyst recycling without any loss. Also, CS/Ag bionanocomposites could be used as a catalyst for the removal of heavy metals including (Cd, Pb, Cu, and Fe) by adsorption technique. Additionally, chitosan and Ag nanoparticles mixture showing antimicrobial activity against gram-negative bacteria like (*E. coli*) and gram-positive bacteria like (*G. bacillus*). The antimicrobial activity of the mixture is more pronounced for the gram-negative bacteria than that of the gram positive one.

## Declarations

### Author contribution statement

Ahmed Youssef, Alsayed Al-sherbini, Amr Aboelella Hussein: Conceived and designed the experiments; Performed the experiments; Analyzed and interpreted the data; Contributed reagents, materials, analysis tools or data; Wrote the paper.

Hala Ghannam, Gamal El-Ghannam: Conceived and designed the experiments; Analyzed and interpreted the data; Wrote the paper.

### Funding statement

This research did not receive any specific grant from funding agencies in the public, commercial, or not-for-profit sectors.

### Competing interest statement

The authors declare no conflict of interest.

### Additional information

No additional information is available for this paper.
